# Phylomitogenomics supports *Actias isabellae* (Graells, 1849) as the definitive scientific name of the Spanish Moon Moth (Lepidoptera, Saturniidae)

**DOI:** 10.1007/s10709-025-00231-w

**Published:** 2025-03-24

**Authors:** Daniel García-Souto, Sonia Zumalave, Juan M. Martínez-Romero, Neus Marí-Mena, Antón Vizcaíno, Marta Vila

**Affiliations:** 1 Genomes and Disease, Centre for Research in Molecular Medicine and Chronic Diseases (CIMUS), Universidade de Santiago de Compostela, Avenida Barcelona, 15782 Santiago de Compostela, Spain; 2 Centro de Investigación Mariña, Universidade de Vigo, Vigo, Spain; 3 National Biotechnology Center| CSIC, Campus Cantoblanco, Universidad Autónoma de Madrid, Calle Darwin 3, 28049 Madrid, Spain; 4 AllGenetics and Biology SL, Rúa Cubelos 21, 15172 Oleiros, A Coruña, Spain; 5 Grupo de Investigación en Bioloxía Evolutiva (GIBE), Departamento de Bioloxía, Facultade de Ciencias, Universidade da Coruña, Campus da Zapateira, 15071 A Coruña, Spain

**Keywords:** Hybrid assembly, Illumina, Mitogenome, NGS, ONT, Zoological nomenclature

## Abstract

The taxonomic classification of the Spanish Moon Moth has been contentious for over a century, with debates over its placement within the genera *Graellsia* and *Actias*. This study presents a comprehensive analysis of the complete mitochondrial genome (mitogenome) of this iconic insect, revealing a closed circular molecule of 15,252 bp containing 37 genes, consistent with the mitochondrial genomes of other Lepidoptera. Phylomitogenomic analyses confirm that the Spanish Moon Moth clusters monophyletically with *Actias dubernardi* and the other species of this genus, supporting the assertion that *Graellsia* is a junior synonym of *Actias*. Our findings further highlight that the shared ancestry of these species suggests a common evolutionary origin for the pine-feeding trait, challenging previous notions of parallel evolution. The implications of this taxonomic revision are significant, as *Actias isabellae* is protected under various European conservation laws. This research provides the crucial genetic data necessary for the formal recognition of *Actias isabellae*, potentially prompting updates to legal classifications and enhancing our understanding of Lepidopteran biodiversity.

## Introduction

The taxonomic placement of the Spanish Moon Moth, an iconic species of the European fauna, has intrigued entomologists for over a century. Although mainly referred to as *Graellsia isabellae* (Graells 1849) (e.g. Marí-Mena et al. 2019), a growing number of publications use the scientific name *Actias isabellae* (Graells 1849) (e.g. Breton et al. 2018; Naumann and Lien 2024) since the seminal work of Nässig (1991), who suggested *Graellsia* to be a junior subjective synonym of *Actias.* That proposal was based on the available knowledge about morphology, host-plant preferences, and phylogenetics of the group at that time. Subsequent research further reinforced the maintenance of genus *Graellsia* based on phylogenetic analysis of several nuclear regions, as well as some behavioural and morphological traits (Regier et al. 2002; Ylla et al. 2005; Boulet-Audet et al. 2015). However, recent phylogenomic works based on nuclear information put forward *Actias isabellae* as the correct scientific name (Rougerie et al. 2015, 2022), leading us to revisit the nomenclature and taxonomic status of the Spanish Moon Moth from the mitogenomic perspective.

The taxonomic assignment of the Spanish Moon Moth holds significance beyond a purely academic question. This lepidopteran is protected under the name *Graellsia isabellae* by the Bern Convention (Appendix III; Council of Europe 1979) and the Habitats Directive of the European Union (Annexes II and V; Council of the European Union 1992). Consequently, it is also protected in France (Ministère de l’Écologie et du Dévelopment Durable 2007) and Spain (Ministerio de Medio Ambiente y Medio Rural y Marino 2011), listed under the genus *Graellsia* in both documents and their regional derivatives. Should the scientific name finally be changed to *Actias isabellae*, many legal documents will have to be updated.

In this study, we firstly present a comprehensive analysis of the mitogenome of the Spanish Moon Moth. Then, we aim to clarify its evolutionary position within regard to the genus *Actias* by means of phylomitogenomics. We thus provide the last piece of genetic information needed to establish the correct scientific name of the only European member of the spectacular “Moon Moths”.

## Materials and methods

### Sampling, DNA extraction and NGS sequencing

We extracted genomic DNA from the thorax of an adult female collected on May 26th, 2009 at the Pyrenees (Montferrer, Lleida, Spain; 42º20’N 1º25’E) and kept at – 20ºC until analysis. We used the NZYTissue gDNA isolation kit (NZYTech), strictly following the manufacturer’s instructions. DNA was resuspended in a final volume of 100 µL. The resulting DNA concentration, 76 ng/µL was measured using the Qubit dsDNA High Sensitivity Assay (Thermo Fisher Scientific).

We aimed at a hybrid assembly combining Illumina short reads and Nanopore long reads. Therefore, a first genomic DNA library was prepared from the extracted DNA, using the Illumina TruSeq Nano kit, strictly following the manufacturer’s instructions, aiming for a target mean insert size of 350 bp. The library was dual-indexed for post-sequencing demultiplexing and sequenced in a fraction of a HiSeqX PE150 lane, for a total output of 50 Gigabases. Postprocessing included a quality check using FASTQC v 0.12.1 (Andrews 2010) and removal of adapter sequences and low-quality reads using TRIMMOMATIC v 0.39 (Bolger et al. 2014).

Given the quality of the genomic DNA used for Illumina sequencing, we proceeded with a new extraction from the same individual using the MagAttract HMW DNA Kit (Qiagen) in order to obtain longer fragments. The kit was used according to the manufacturer’s instructions, except that the amount of tissue and reagents used were scaled up by a factor of three in order to obtain enough quantity of DNA for library preparation. The isolated DNA was further purified by solid-phase reverse immobilization (SPRI) using the MagBind RxnPure Plus magnetic beads (Omega Biotek) and the Short Read Eliminator XS Kit (Circulomics) following the manufacturer recommendations in order to remove shorter fragments. The integrity of the isolated and purified DNA was checked with a TapeStation genomic DNA ScreenTape (Agilent Technologies), while the concentration was measured using the Qubit brDNA Assay (Thermo Fisher Scientific), yielding a DIN value of 8.8 and a final concentration of 60 ng/µL.

A total amount of 2.6 µg of the high molecular weight unsheared DNA sample was used as input for two Oxford Nanopore Technologies (ONT) libraries. In brief, following end-repairing and dA-tailing using the end-prep repair (NEBNext FFPE DNA Repair Mix, New England BioLab) and adapter ligation (NEBNext Ultra II Ligation Module, New England BioLab) modules, whole-genome libraries were constructed with the Oxford Nanopore Sequencing 1D ligation library prep kit (SQK-LSK108, Oxford Nanopore Technologies) following the manufacturer recommendations. Genomic libraries were loaded onto MinION R9.4 flowcells in MinION devices controlled by MinKNOW (v19.12.5). Both libraries run for 78 h until complete exhaustion, and the basecaller GUPPY 6.5.7 (Oxford Nanopore Technologies 2018) was employed to generate fastq files in super accuracy mode.

### Mitogenome assembly

We successfully assembled the complete mitochondrial genome of the Spanish Moon Moth following a hybrid strategy by combining Illumina short reads and Nanopore long reads. In brief, we extracted a subset of mitochondrial reads from both Illumina and Nanopore sequencing datasets based on their similarity with established reference mitogenomes from the Saturniidae family available at Genbank as of November 9th, 2023 (Table 1). We decided not to include the mitogenome of *Actias maenas* (MG836834) in our dataset, as that record appears as “Unverified”. In addition, the mitogenomes of *Saturnia (Neoris) haraldi* (NC_036765) and *Cricula trifenestrata* (KY644697) were excluded from the final dataset, as our preliminary phylogenetic analyses pointed to either misidentifications or substandard sequencing quality, as previously suggested by Nethavhani et al. (2022) and Liu (2024).

We mapped the Nanopore and Illumina reads onto the existing mitogenomes using BWA-mem v0.7.17-r1188 (Li 2013) and minimap2 v2.24 (Li 2018), respectively. Subsequent steps included sorting, quality filtering (applying a threshold of -q60) and conversion to fastq format utilizing samtools v1.14 (Li et al. 2009) and picard v2.25.5 (https://broadinstitute.github.io/picard/). Nanopore reads exceeding 25 Kb, attributable to possible nuclear mitochondrial DNA inserts (NUMTs), were discarded using seqtk v1.3 (https://github.com/lh3/seqtk).

**Table 1:** Accession numbers and nomenclature details of the 42 mitogenomes retrieved from GenBank

Species	Accession number	Species name as in GenBank	References
*Actias aliena*	KF927042	*Actias artemis aliena*	Park et al. (2014)
*Actias dubernardi*	MW133617		
*Actias luna*	NC_045899		
*Actias selene*	NC_018133		
*Antheraea assamensis*	NC_030270	*Antheraea assama*	Paukstadt et al. (2000)
*Antheraea assamensis*	KU379695		
*Antheraea formosana*	OK078922		
*Antheraea frithi*	KJ740437		
*Antheraea pernyi*	AY242996		
*Antheraea pernyi* x *A. roylei*	NC_044744		
*Antheraea polyphemus*	NC_072344		
*Antheraea yamamai*	NC_012739		
*Attacus atlas*	NC_021770		
Bombyx mandarina*	NC_003395		
*Bunaea alcinoe*	NC_061295		
Clanis deucalion*	MT712135		
*Eochroa trimenii*	NC_061324		
*Epiphora bauhiniae*	NC_061325		
*Gonimbrasia belina*	NC_046032		
*Gonimbrasia tyrrhea*	NC_061326		
*Gynanisa maja*	NC_046033		
*Heniocha apollonia*	NC_061296		
*Heniocha dyops*	NC_061327		
*Holocerina smilax*	NC_061297		
*Ludia delegorguei*	NC_061328		
Marumba sperchius*	NC_067721		
*Nudaurelia cytherea*	NC_061331	*Gonimbrasia cytherea*	Nethavhani et al. (2022)
*Nudaurelia wahlbergi*	NC_061330		
*Rhodinia fugax*	MT548575		
*Rinaca boisduvalii*	NC_010613	*Saturnia boisduvalii*	Rubinoff and Doorenweerd (2020)
*Rinaca japonica*	NC_063568	*Saturnia japonica*	Rubinoff and Doorenweerd (2020)
*Rinaca jonasii*	MF346379		
*Salassa thespis*	OR522707		
*Samia canningi*	NC_024270		
*Samia cynthia*	KC812618	*Samia cynthia cynthia*	Peigler and Naumann (2003)
*Samia ricini*	NC_017869	*Samia cynthia ricini*	Peigler and Naumann (2003)
*Samia wangi*	NC_068878		
*Samia watsoni*	NC_068851		
*Saturnia pavonia*	OX383924		
*Saturnia pyretorum*	FJ685653	*Eriogyna pyretorum*	Naumann and Löffler (2005)
*Vegetia ducalis*	NC_061298		
*Vegetia grimmia*	NC_061329		

The three non-Saturniidae species used as outgroups are marked with asterisks. ‘Reference’ indicates the scientific literature supporting the species name if it differs from the one recorded in GenBank

Following this, we constructed a preliminary draft genome using mitoVPG v2.2 (Formenti et al. 2021) using the above long and short mitochondrial datasets and priming the assembly with the mitochondrial genome reference from a related species (*Actias luna*, MN832537.1). The resulting primary assembly underwent a polishing step to rectify potential artifacts. To do so, Illumina reads were remapped using BWA-mem v0.7.17-r1188 (Li 2013), with resulting alignments sorted, quality-filtered (-q60) and indexed using samtools v1.10 (Li et al. 2009). Two rounds of polishing were then conducted using Pilon v1.23 (Walker et al. 2014), and the accuracy of single nucleotide variants (SNVs) was assessed through visual inspection of the mapped paired-end reads using the Integrative Genomics Viewer (IGV) v2.13.1 (Robinson et al. 2011). To evaluate potential long structural artifacts, Nanopore reads were re-mapped onto the draft assembly using minimap2 v2.24 (Li 2018), followed variant calling with sniffles v2.2 (Sedlazeck et al. 2018) and visual inspection using IGV.

### Mitogenome annotation

For genome annotation, we employed the MITOS2 web server (Donath et al. 2019, http://mitos.bioinf.uni-leipzig.de/) to trace and annotate all protein coding genes, rDNAs and tDNAs within the corrected mitochondrial genome. The annotation process adhered to the mitochondrial genetic code for invertebrates (transl_table = 5) and the RefSeq63 dataset for metazoans served as the reference data. To ensure accuracy, we conducted a manual verification of the resulting annotations, matching them with the open reading frame (ORF) predictions obtained by ORF-FINDER (Rombel et al. 2002). Additionally, we cross-referenced the annotations with known transcriptional exceptions documented for the Saturniidae mitogenomes available in GenBank (Table 1). To do so, we conducted a multiple alignment with CLUSTALW (Thompson et al. 1994) as implemented in GENEIOUS v11.0.9 + 11 (www.geneious.com) between the assembly obtained with the rest of the species to detect incorrectly annotated 5’ gene starts, due to alternative codon starts, and possible 3’ overhangs or abrupt ends, due to alternative stop codons. The visualization and graphing of the data were performed with the Proksee website (https://proksee.ca/, Grant et al. 2023).

### Phylogenomic analysis

We aligned the 13 protein coding genes (PCGs) of the newly obtained mitogenome and the ones of *Saturnia pavonia* (OX383924, not annotated) with those of the other 38 available Saturniidae species (Table 1) and three other Lepidoptera selected as outgroups: two members from the Sphingidae family (*Marumba sperchius* and *Clanis deucalion*), which is the sister group of Saturniidae (Hamilton et al. 2019), and one member of Bombycidae (*Bombyx mandarina*) (Table 1). The alignment of the 13 PCGs was performed with MUSCLE v 3.8.425 (Edgar 2004), resulting in a total length of 11,364 bp.

A Bayesian phylogenetic analysis (BI) of the 43 mitogenomes was conducted using MRBAYES 3.2.6 (Huelsenbeck and Ronquist 2001). Each PCG was analysed with its respective substitution model, which was selected based on the AIC criterion using JMODELTEST2 (Guindon and Gascuel 2003; Darriba et al. 2012). The analysis utilized a Markov Chain Monte Carlo (MCMC) approach with four chains, running for 100,000 generations with samples taken every 200 generations. *Bombyx mandarina* (FJ384796) was designated as the outgroup, and the analysis was initiated with a random starting tree. To account for burn-in, the initial 5,000 trees from each chain were discarded, and the consensus tree with posterior probabilities was summarized.

We also calculated a Maximum Likelihood (ML) phylogeny for the 43 mitogenomes using PHYML 3.0 (Guindon et al. 2010). We used the default settings except for the AIC selection criterion (automatic model selection by SMS; Lefort et al. 2017) and the estimation of branch support (1,000 standard bootstrap replicates).

## Results

**Fig. 1:**
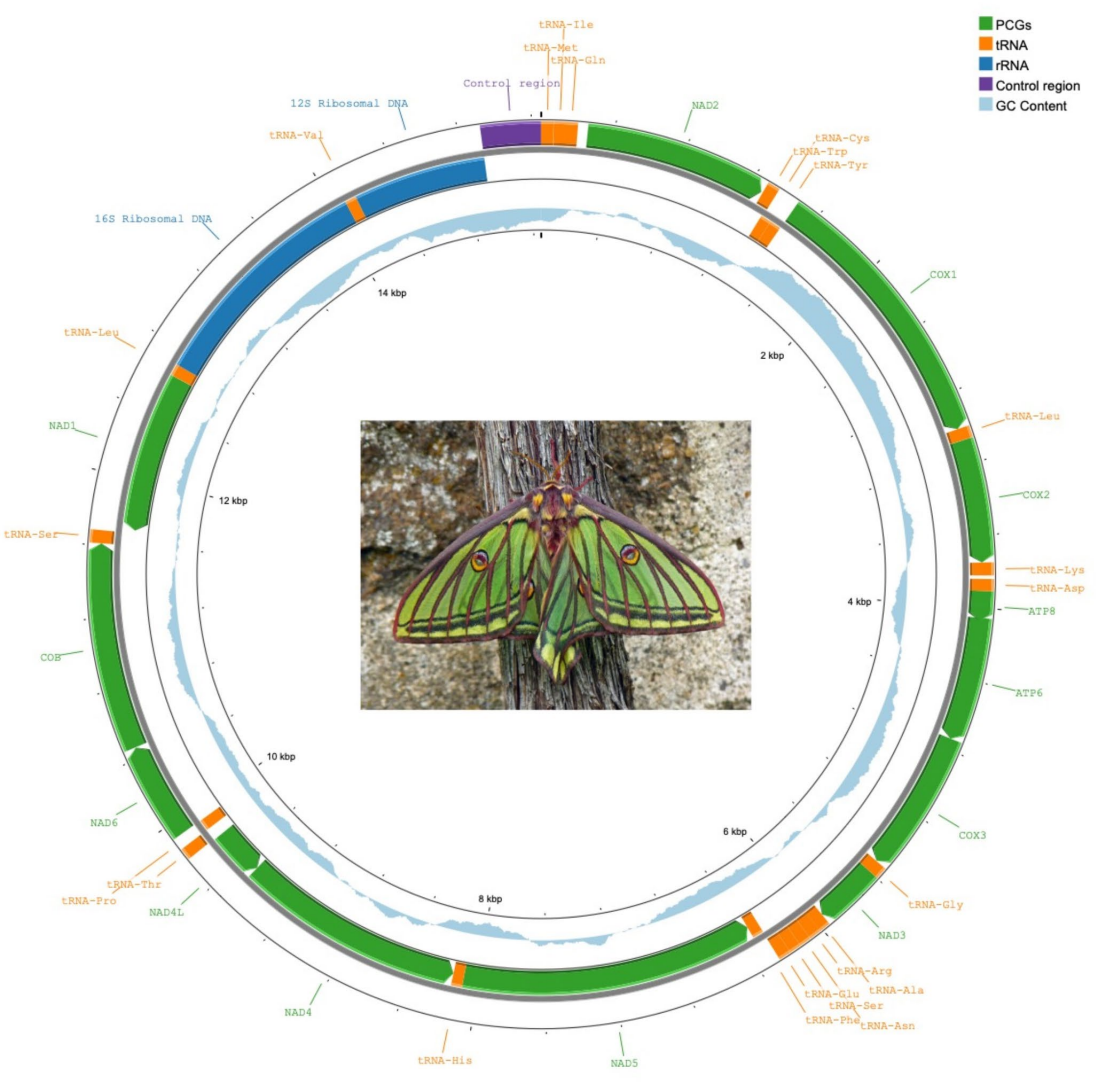
Gene map of the mitochondrial genome of the Spanish Moon Moth (15,252 bp). The circular map was generated using Proksee, illustrating the arrangement and organization of mitochondrial genes in this species. The inner circle shows the GC skew. The inset displays an adult female (image by J-Y-M, licensed under CC BY-NC-ND 3.0, https://creativecommons.org/licenses/by-nc-nd/3.0/)

The complete mitogenome of the Spanish Moon Moth is a closed circular molecule, 15,252 bp in length. It contains the usual metazoan set of 37 genes (13 PCGs, 22 tRNAs, 2 rRNAs) and a control region (Fig. 1). The latter is usually known in insects as A + T rich region and it was 330 bp in length in our study specimen (A + T composition = 90.6%). The overall base composition was 39.1% A, 40.4% T, 12.5% C, and 8.0% G. All of the PCGs used the standard start codon ATN, except for *COX1* that starts with CGA (Table 2). The newly obtained mitogenome of the Spanish Moon Moth was deposited in GenBank under the accession number OR790126.

**Table 2:** Gene order and organization of the mitochondrial genome of the Spanish Moon Moth

Gene	Strand	Location	Size (bp)	Anticodon	Start codon	End codon
tRNA-Met	J	1–68	68	cat		
tRNA-Ile	J	70–133	64	gat		
tRNA-Gln	N	131–199	69	ttg		
ND2	J	256–1269	1014		ATT	TAA
tRNA-Trp	J	1278–1346	69	tca		
tRNA-Cys	N	1339–1401	63	gca		
tRNA-Tyr	N	1402–1467	66	gta		
COX1	J	1470–3000	1531		CGA	T*
tRNA-Leu	J	3001–3066	66	taa		
COX2	J	3068–3749	682		ATG	T*
tRNA-Lys	J	3750–3820	71	ctt		
tRNA-Asp	J	3840–3905	66	gtc		
ATP8	J	3906–4070	165		ATT	TAA
ATP6	J	4055–4741	687		TTG	TAA
COX3	J	4741–5529	789		ATG	TAA
tRNA-Gly	J	5532–5598	67	tcc		
ND3	J	5599–5952	354		ATT	TAG
tRNA-Ala	J	5951–6016	66	tgc		
tRNA-Arg	J	6016–6081	66	tcg		
tRNA-Asn	J	6082–6146	65	gtt		
tRNA-Ser	J	6147–6212	66	gct		
tRNA-Glu	J	6213–6280	68	ttc		
tRNA-Phe	N	6279–6343	65	gaa		
ND5	N	6344–8087	1744		ATT	T*
tRNA-His	N	8088–8152	65	gtg		
ND4	N	8153–9493	1341		ATG	TAA
ND4L	N	9494–9784	291		ATG	TAA
tRNA-Cys	J	9791–9855	65	tgt		
tRNA-Pro	N	9856v9920	65	tgg		
ND6	J	9923–10,459	537		ATA	TAA
COB	J	10,462–11,610	1149		ATA	TAA
tRNA-Ser	J	11,616–11,682	67	tga		
ND1	N	11,700–12,638	939		ATG	TAG
tRNA-Leu	N	12,640–12,707	68	tag		
lrRNA (16 S)	N	12,708–14,077	1370			
tRNA-Val	N	14,078–14,143	66	tac		
srRNA (12 S)	N	14,144–14,922	779			

J (+) or N (–) indicates gene directions

*Stands for incomplete stop codon terminated by the addition of an AA dinucleotide during mRNA processing

**Fig. 2:**
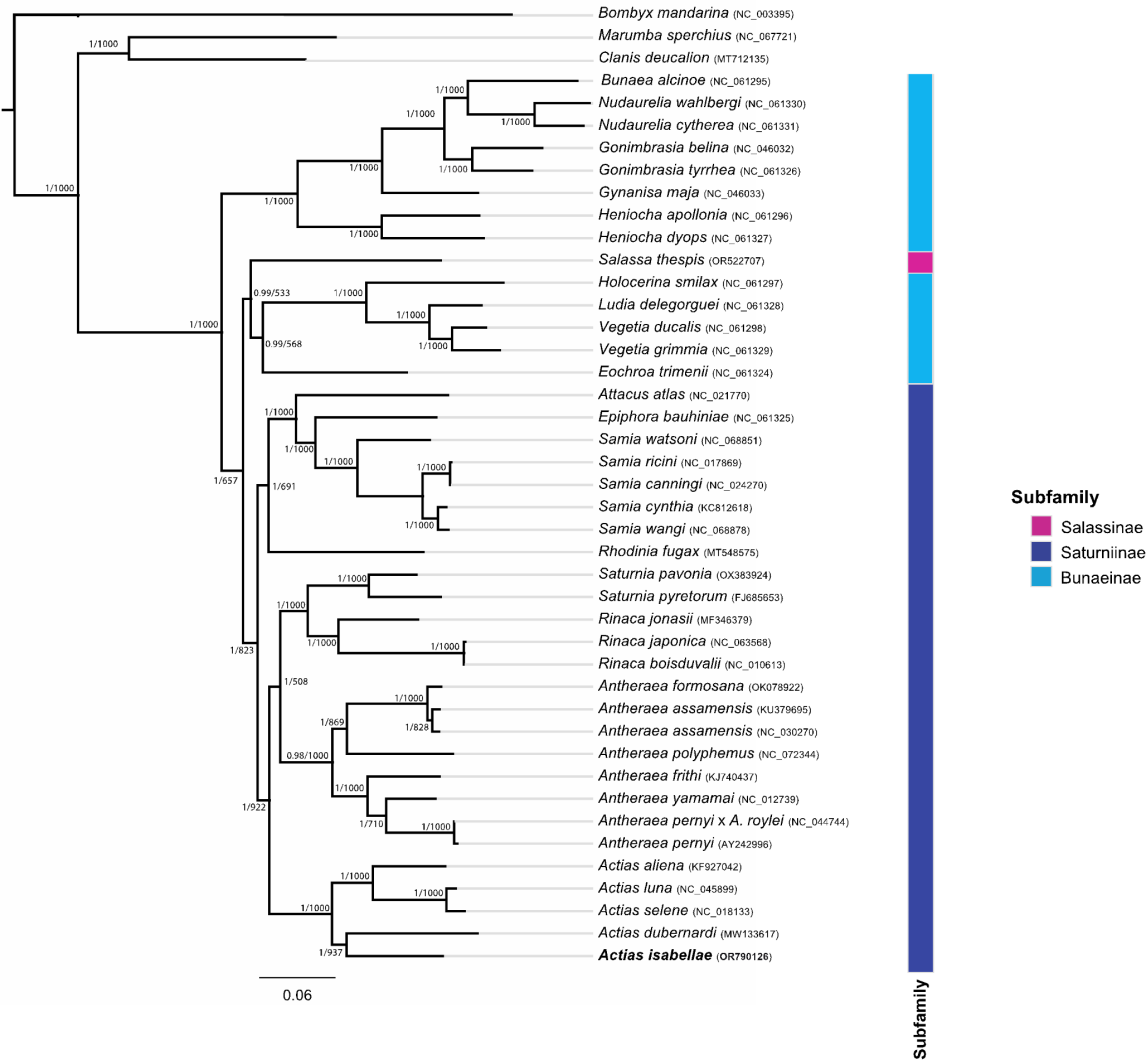
Bayesian inference (BI) phylogenetic tree based on the mitochondrial genomes of 39 Saturniidae species and three outgroups. Branch support is shown in the following format: posterior probabilities (0–1) from the Bayesian phylogeny, followed by a forward slash, and then bootstrap values from the ML reconstruction

Phylogenetic analyses, based on 13 PCGs of 39 complete mitogenomes (ingroup) and three outgroups, unambiguously clustered all congeneric species into well supported monophyletic groups (Fig. 2). Bayesian and Maximum Likelihood reconstructions yielded identical topologies. The mitogenomes of the Spanish Moon Moth and the four species of genus *Actias* were recovered as a unique monophyletic cluster with maximal node support in all analyses.

## Discussion

The size and structure of the Spanish Moon Moth mitogenome was similar to those of most Lepidoptera (e.g. Zhao et al. 2021 and references therein; Zhu et al. 2023; Liu 2024). As expected, its nucleotide composition was biased toward A + T (78.2%).

The analysed mitogenomes unambiguously grouped congeneric species into well-supported monophyletic groups. The mitogenomes of the Spanish Moon Moth and the four *Actias* species formed a highly supported clade, providing additional evidence in favour of the name *Actias isabellae*, as previously suggested by Nässig (1991) and supported by nuclear phylogenomic analyses (Rougerie et al. 2022). We therefore confirm that genus *Graellsia* Grote, 1896 should be recognised as a junior synonym of *Actias* Leach, 1815.

The phylogenetic topology we recovered for the five species of Moon Moths is congruent with nuclear genomic results obtained by Rougerie et al. (2015) and Rubin et al. (2024). Specifically, our mitochondrial results confirm that *A. isabellae* and *Actias dubernardi* belong to the conifer-feeding *Actias* cluster, a well-supported monophyletic group (Naumann and Lien 2024), whereas *Actias aliena*, *Actias luna*, and *Actias selene* are classified within the separate clade of angiosperm specialists. These findings further refute that hypothesis that parallel evolution explains the Spanish Moon Moth larvae’s adaptation to fine feeding, as initially suggested by Nässig (1991).

Lastly, *Graellsia* should not be considered a subgenus of *Actias* either. Subgenera are typically defined to provide clarity when a genus contains high diversity, distinct evolutionary lineages, or notable morphological or ecological differences among species (e.g. Naumann et al. 2012). While *A. isabellae* is biogeographically distinct as the only *Actias* species naturally occurring in Europe, genomic evidence clearly places it within the monophyletic group of conifer-feeding *Actias*. Despite the historical significance of the genus *Graellsia* in the entomological literature, we argue that establishing a subgenus is not warranted. Instead, recognising the Spanish Moon Moth as *A. isabellae* maintains taxonomic consistency and facilitates communication within the scientific community.

## Conclusions

Our phylogenetic analyses confirm the monophyly of the genus *Actias*, with the Spanish Moon Moth clustering alongside *Actias dubernardi*. This finding supports previous recommendations to retain the name *Actias isabellae* and challenges the distinction of *Graellsia* as a subgenus.

## Data Availability

The newly obtained mitogenome of the Spanish Moon Moth was deposited in GenBank under the accession number OR790126.
